# A universal similarity based approach for predictive uncertainty quantification in materials science

**DOI:** 10.1038/s41598-022-19205-5

**Published:** 2022-09-02

**Authors:** Vadim Korolev, Iurii Nevolin, Pavel Protsenko

**Affiliations:** 1 Department of Chemistry, Lomonosov Moscow State University, Moscow, 119991 Russia; 2 Frumkin Institute of Physical Chemistry and Electrochemistry, Russian Academy of Sciences, Moscow, 119071 Russia

**Keywords:** Computational chemistry, Cheminformatics, Computational methods

## Abstract

Immense effort has been exerted in the materials informatics community towards enhancing the accuracy of machine learning (ML) models; however, the uncertainty quantification (UQ) of state-of-the-art algorithms also demands further development. Most prominent UQ methods are model-specific or are related to the ensembles of models; therefore, there is a need to develop a universal technique that can be readily applied to a single model from a diverse set of ML algorithms. In this study, we suggest a new UQ measure known as the Δ-metric to address this issue. The presented quantitative criterion was inspired by the *k*-nearest neighbor approach adopted for applicability domain estimation in chemoinformatics. It surpasses several UQ methods in accurately ranking the predictive errors and could be considered a low-cost option for a more advanced deep ensemble strategy. We also evaluated the performance of the presented UQ measure on various classes of materials, ML algorithms, and types of input features, thus demonstrating its universality.

## Introduction

Supervised machine learning (ML) has tremendously transformed the modeling of structure–property relationships^1–3^. Cutting-edge studies have addressed the implementation of new featurization schemes, such as materials representations^4–7^ and the adaptation of neural network architectures to domain-specific input data (crystal structures^8–10^ and chemical compositions^11–13^). In parallel, the materials informatics community has created a diverse suite of user-friendly packages covering different stages of the ML pipeline^14–21^. A sharp increase in the number of predictive algorithms, materials representations, and applications has driven the development of benchmarking protocols and data sets^22–24^. However, another aspect of using ML, namely, uncertainty quantification (UQ)^25,26^, has received much less attention, although it has also been crucial to the exploration of subdomains of materials spaces that significantly differ from the training set. Thus, advanced materials informatics tasks, such as materials discovery, have been shown to be most sensitive to this issue^27–30^.

Most studies have focused on the applicability of predictive models and reliability of individual outputs (prediction intervals) to perform UQ using universal, but cost-prohibitive, variational-inference-based methods, such as Monte Carlo dropout^31,32^, bootstrapping/subsampling^29,31,33,34^, and deep ensembles^31^. Gaussian process regression (GPR)^35–37^ is an alternative approach that intrinsically provides the output and associated uncertainty. Beyond these well-known straightforward solutions, only a few techniques have been adopted for materials informatics. In particular, Janet et al.^38^ introduced the distance to training data in the latent space of a neural network as a low-cost UQ metric, and predictive error decreased systematically by tightening the threshold of this parameter. In addition, Sutton et al.^39^ presented a tool based on subgroup discovery. The conditioned combinations of structural and compositional features derived by the method defined the subdomains of a materials space with a model error that was lower than average for all considered materials.

A closely related field to materials informatics, chemoinformatics, has also been confronted with the problem of UQ. For example, three main groups of algorithms have been adapted for ML-assisted drug design^40^. First, frequentist methods drew the statistical inference only from the likelihood without a prior hypothesis, and the UQ for the molecular property prediction tasks was provided^41–44^. Second, Bayesian approaches have been successfully utilized for the same purpose^45–50^. The third group of methods, known as empirical techniques, have relied on the concept of applicability domain^51–55^. Generally, this can be expressed as a chemical structure (descriptor) subspace where the predictive model provides a reliable output. Thus, UQ methods involving applicability domain estimation do not consider information from the approximation algorithm. Consequently, the universality of model-agnostic methods interacts with the simple assumption that their performance is only determined by the distribution of training points in chemical space.

In this study, we have presented a new UQ measure, Δ-metric, based on ideas of applicability domain estimation originated from chemoinformatics, and the remainder of this manuscript describes the principles of its construction and benchmarking results. We considered four use cases of bandgap prediction to assess the efficacy of the Δ-metric in ranking predictive errors and calculating predictive intervals. The performance of the suggested metric in UQ was compared to the performance of widespread methods, including deep ensembles, subsampling, and the infinitesimal jackknife variance. In addition, two out-of-domain use cases were also discussed. The provided results depicted a set of scenarios where the Δ-metric would be a potentially helpful UQ technique.

## Methods

### Data sets

For this study, 10,434 inorganic crystal structures and their corresponding bandgap values were obtained from the computational database presented by Kim et al.^56^ Initially, the band edges were identified within the generalized gradient approximation, as implemented by Perdew, Burke, and Ernzerhof (PBE)^57^. Then, one-shot calculations with the screened hybrid functional of Heyd, Scuseria, and Ernzerhof (HSE06)^58^ were conducted to estimate the bandgap values at the *k* points of the band edges found with the PBE functional. Structures containing noble gases were excluded from consideration. Three hundred fifty-eight two-dimensional materials and their corresponding bandgap values were collected from the Computational 2D Materials Database^59–61^. We considered structures with bandgaps that were calculated within three available levels of theory: PBE, hybrid HSE06, and many-body GW approximation. A total of 14,204 MOF structures and their corresponding PBE bandgap values were obtained from the Quantum MOF database^62^, and 12,500 molecular crystals and their corresponding PBE bandgap values were obtained from the Organic Materials Database^63,64^.

### Feature extraction and preprocessing

For the MEGNet^9^ model, we used elemental embeddings from the original study trained on the formation energy, which was kept fixed during our model training. The CFIDs^65^, as implemented in the matminer^17^ package, were used to featurize the two-dimensional materials. The thirty CFIDs with the highest F-values were selected for further consideration, and the PBE bandgap was incorporated as a crude estimator of the GW bandgap^66–68^. The StandardScaler was applied to normalize the above features. The attributes proposed by Meredig et al.^69^ (atomic fractions of elements and statistics of elemental properties), as implemented in the matminer^17^ package, were used to featurize the MOFs, and the MinMaxScaler was implemented to normalize these features. We used the PLMFs proposed by Isayev et al.^7^ to featurize the molecular organic materials, and specifically, the linear chains up to four atoms and the first shell of the nearest-neighbor atoms were considered. Only atomic numbers were taken into account to label local fragments. Atomic connectivity was defined according to a distance criterion with a tolerance of 0.25 Å. In contrast to the original study, the Voronoi–Dirichlet partition was not applied for reasons of simplicity. The PLMFs normalized per Å^3^ were processed by VarianceThreshold, where features with a training-set variance greater than 10^−7^ were chosen for the following consideration. The selected features were normalized using the MinMaxScaler. All the data were prepared within the scikit-learn^70^ processing routines.

### Machine learning and uncertainty quantification models

The GPR, KRR, and RF models implemented in the scikit-learn^70^ library were trained on 80% of the data and tested with the remaining 20%. A train-validation-test split of 80%–10%–10% was applied for the MEGNet^9^ model. For the GPR model, we used a sum-kernel including the radial-basis function and rational quadratic kernels, where the noise level was set to 1.0 via the $\alpha$ parameter. The KRR model was trained using the Laplacian kernel, with a regularization strength $\alpha$ of 0.1, and parameter $\gamma$ of 0.1. The RF model was trained using 1000 trees, and all other hyperparameter values of the above models were set to the default. The MEGNet model was trained using the hyperparameter values proposed in the original study.

The SOAP-like descriptor for the Δ-metric was constructed using the DScribe^18^ library. The number of radial basis functions ${n}_{max}$ and maximum degree of spherical harmonics ${l}_{max}$ were set to eight and six, respectively, while the degree $\zeta$ in Eq. (3) was set to four. Quantile regression analysis was performed using the statsmodels^71^ package.

## Results and discussion

### Definition of the UQ metric

The presented UQ measure, Δ-metric, was deeply inspired by the *k*-nearest neighbor approach adopted for applicability domain evaluation. Specifically, the average distance to the *k* closest training set points was compared to the pre-defined threshold in the most widespread formulation^72^. According to the original definition of the weighted *k*-nearest neighbor algorithm, we proposed the following formula for the $i$-th structure in the test set:

1 $$\begin{array}{*{20}c} {{\Delta }_{i} = \frac{{\mathop \sum \nolimits_{j} K_{ij} \left| {\varepsilon_{j} } \right|}}{{\mathop \sum \nolimits_{j} K_{ij} }} ,} \\ \end{array}$$

where $\varepsilon_{j}$ is the error between the true and predicted target values of the $j$-th neighbor structure in the training set, and $K_{ij}$ is the corresponding weight coefficient. It was natural to represent $K_{ij}$ as a similarity measure between the *i*-th and *j*-th structures. For this purpose, we implemented a kernel proposed by Bartok et al.^4^, which used the form of a normalized dot product raised to the $\zeta$-th power:

2 $$\begin{array}{*{20}c} {K_{ij} = \left( {\frac{{p_{i} p_{j} }}{{p_{i} p_{j} }}} \right)^{\zeta } ,} \\ \end{array}$$

where $p$ is a global descriptor. To featurize the structures, we used a smooth overlap of the atomic positions (SOAP) descriptor^73^, as given by

3 $$\begin{array}{*{20}c} {p_{{n_{1} n_{2} l}} = \frac{\pi }{{N^{2} }}\sqrt {\frac{8}{2l + 1}} \mathop \sum \limits_{m} \mathop \sum \limits_{i,j} \left( {c_{{n_{1} lm}}^{i} } \right)^{\dag } c_{{n_{2} lm}}^{j} ,} \\ \end{array}$$

where $c_{{n_{1,2} lm}}^{i,j}$ are the expansion coefficients in terms of the radial basis functions (labeled by $n_{1,2}$) and angular momentum channels (labeled by $l$) for the $i,j$-th atom, and $N$ is the number of atoms. Therefore, the Δ-metric calculations required only that the following quantities be obtained from ML model training: the atomic structure of interest, the atomic structures from the training set, and the absolute errors of prediction at these points.

### UQ in bandgap prediction

**Figure 1:**
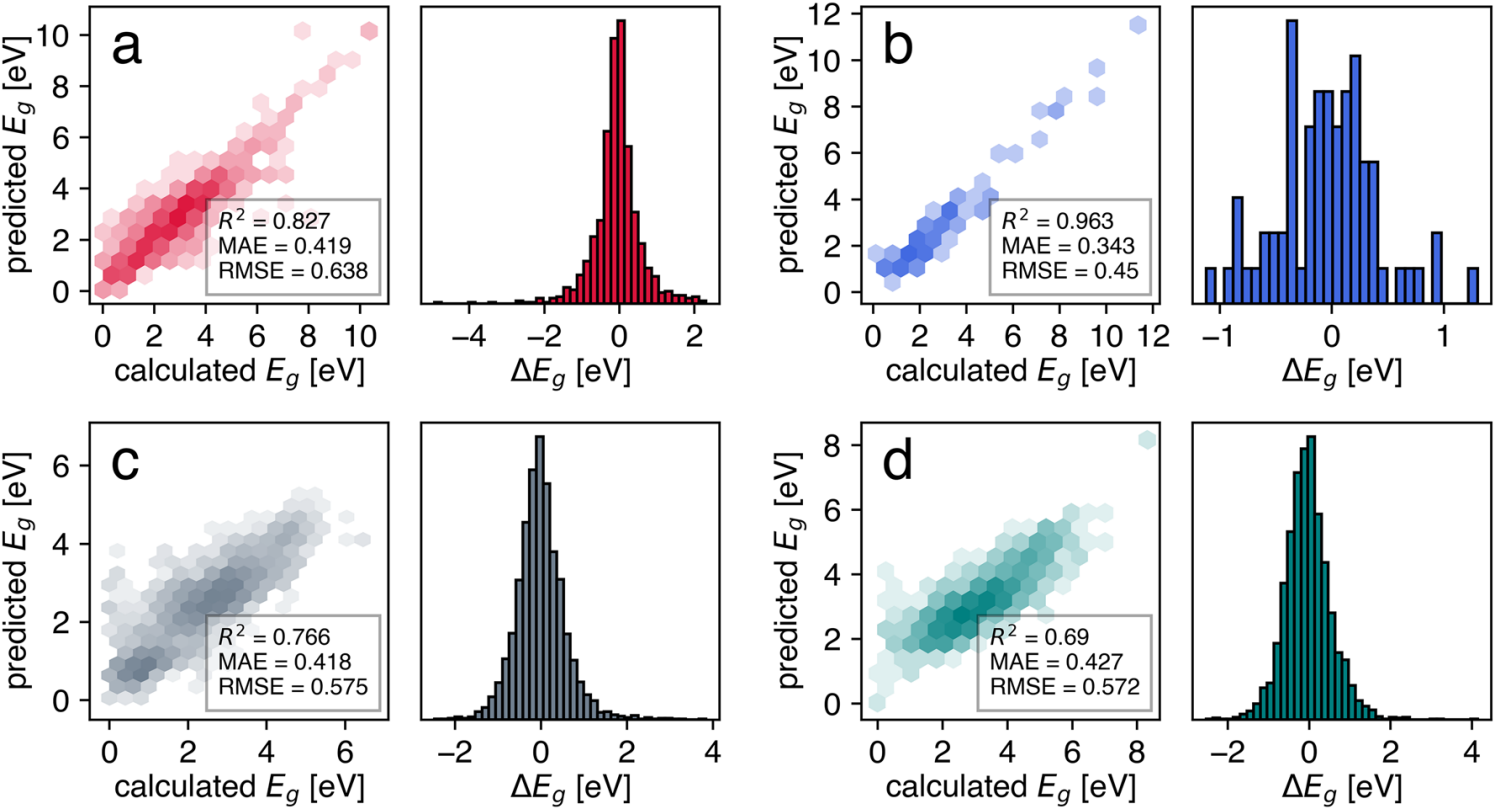
Parity plots and error histograms for the four considered use cases: (**a**) SNUMAT-MEGNet, (**b**) C2DB-GPR-CFID, (**c**) QMOF-KRR-ElemStat, and (**d**) OMDB-RF-PLMF.

To show the efficacy of the proposed UQ metric, we selected bandgap prediction because of its fundamental role in determining material performance in many applications^74–76^. Concretely, four use cases that varied in the predictive model algorithm, type of materials representation, and materials class (the corresponding abbreviations are shown in brackets) were considered: the materials graph network for inorganic crystals (SNUMAT-MEGNet), Gaussian process regression based on classical force-field inspired descriptors for two-dimensional inorganic materials (C2DB-GPR-CFID), kernel ridge regression based on the atomic fractions and statistics of elemental properties for metal–organic frameworks (QMOF-KRR-ElemStat), and random forests based on the fragments of the simplified version of property-labeled materials fragments for organic materials (OMDB-RF-PLMF). A summary of trained model performance is shown in Fig. 1. Although we did not intend to achieve state-of-the-art (SOTA) accuracy in this study, it was still valuable to compare the presented algorithms with those known from the literature. As shown below, these estimates provided a first insight into the ability of the Δ-metric to improve the performance of the predictive model by selecting a specific subset of structures.

Wang et al.^77^ implemented three models to predict the HSE bandgap of inorganic crystals from the SNUMAT database^56^. These models were trained using information from the constituent elements, PBE bandgap, and the combination of inputs from the first two models, respectively. Because we did not use the crude estimator (PBE bandgap) of the target property (HSE bandgap) in the SNUMAT-MEGNet use case, the first model with a root mean squared error (RMSE) of 0.75 eV was considered the SOTA model.

Liang and Zhu^67^ used a set of physicochemical descriptors as inputs for the Lasso algorithm. The model was trained and tested using structures from the C2DB^59,60^ database, reaching a mean absolute error (MAE) of 0.31 eV, which was nearly equivalent to that of our model (0.343 eV). Recently, Satsangi et al.^78^ applied a novel feature selection approach to predict the GW bandgap of two-dimensional materials collected from the C2DB^59,60^ and aNANt repository. The presented GPR model with blended features demonstrated an impressive RMSE of 0.15 eV. However, it should be emphasized that structures from C2DB that belonged only to the two space groups ($P\overline{6}m2$ and $P\overline{3}m1$) were considered. For this reason, we assumed that the results provided by Liang and Zhu^67^ were more relevant for comparison.

Fung et al.^24^ carried out a consistent benchmark study of graph neural networks on several material data sets, including the Quantum MOF database^62^. Several neural networks architectures demonstrated very similar MAE, and slightly outperformed the crystal graph convolutional neural network^8^ model (MAE of 0.274 eV) trained in a study where the MOF data set was presented. Concretely, the SchNet^79^ model had an MAE of 0.228 eV.

Geilhufe and Olsthoorn^80^ applied the same graph neural network architecture to predict the PBE bandgap of the molecular crystals from the OMDB data set, and the model showed an MAE value of 0.406 eV. Olsthoorn et al.^64^ achieved SOTA performance for this task (MAE of 0.388 eV) by averaging the predictions of KRR built on the SOAP kernel and SchNet model.

**Figure 2:**
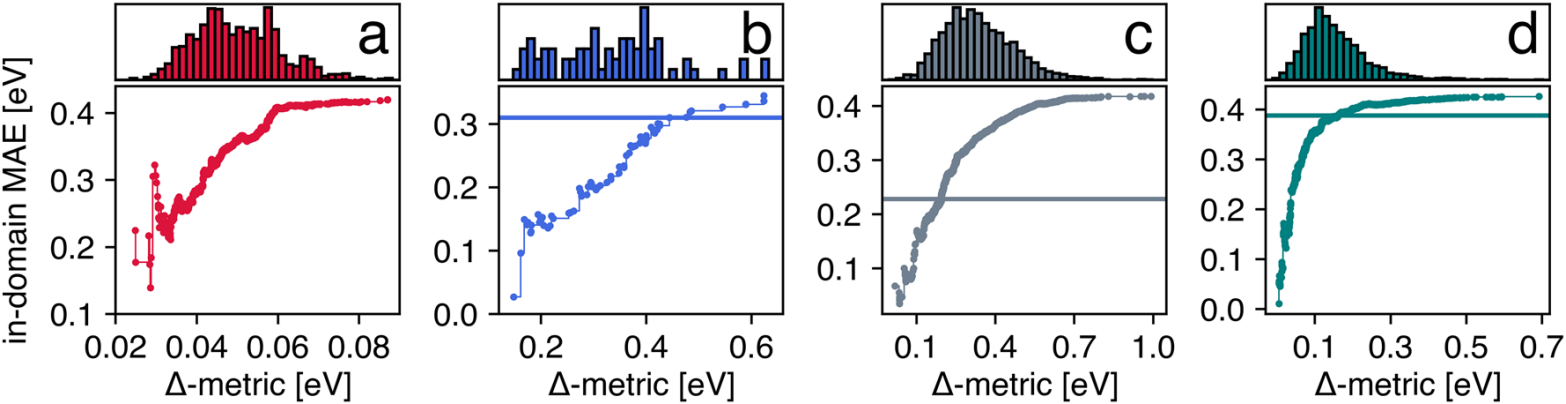
In-domain MAE as a function of the Δ-metric cutoff for the four considered use cases: (**a**) SNUMAT-MEGNet, (**b**) C2DB-GPR-CFID, (**c**) QMOF-KRR-ElemStat, and (**d**) OMDB-RF-PLMF, where the solid horizontal lines indicate the performance of the SOTA models available to date.

As a profitable UQ criterion, the Δ-metric should be able to demarcate the applicability domain of the predictive model. The structure was considered inside the applicability domain if the corresponding Δ-metric value did not exceed the pre-defined threshold, and an increase in model accuracy with a decrease in the threshold was desired. To validate the suggested UQ measure in this context, we examined the in-domain MAE as a function of the Δ-metric cutoff (Fig. 2). Indeed, the most general trend corresponded to the expectation that MAE could be reduced by gradually excluding it from consideration structures with a high Δ-metric value, i.e., high predictive uncertainty. As stated earlier, the performance of the SOTA models for considered tasks appeared to be a reasonable starting point to estimate the significance of decreasing the in-domain MAE. Specifically, the SOTA values of MAE in the C2DB-GPR-CFID, OMDB-RF-PLMF, and QMOF-KRR-ElemStat use cases were achieved at 87.3, 62.3, and 13.2% of the test points with the smallest Δ-metric values, respectively. In the SNUMAT-MEGNet use case, the model implemented in this study entirely covered the applicability domain of the available SOTA algorithm due to the impressive predictive performance of the graph neural network architecture. The results were mainly dependent on the combination of algorithm and material representations that we used, and those that demonstrated SOTA precision; therefore, they should not be considered a universal benchmark of suggested UQ measures. We presented an illustrative example of how Δ-metric helped to extract a (tiny) subspace of structures for which the model built on composition-only features competed on equal terms with the powerful graph neural network (QMOF-KRR-ElemStat use case).

**Figure 3:**
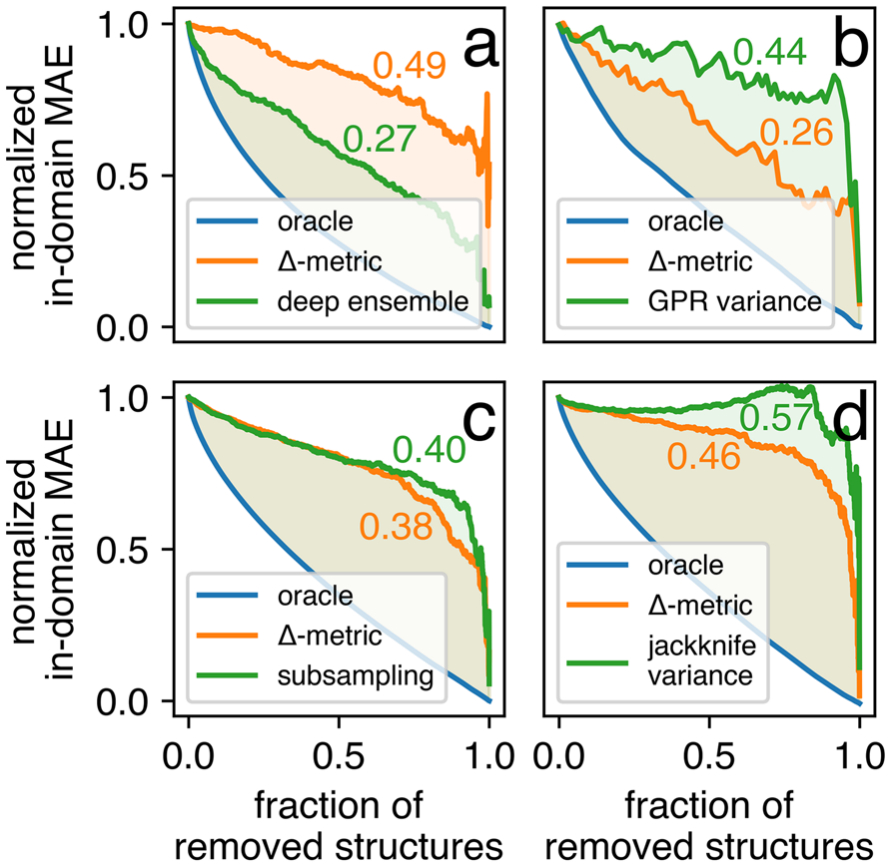
Confidence curves for the four considered use cases: (**a**) SNUMAT-MEGNet, (**b**) C2DB-GPR-CFID, (**c**) QMOF-KRR-ElemStat, and (**d**) OMDB-RF-PLMF. The subgraph includes three cases, the so-called oracle curve (ideal ranking according to observable error), Δ-metric curve (UQ measure presented in this study), and the curve corresponding to the competitive method.

To further explore the efficacy of the Δ-metric, we quantified the sequence monotonicity of the in-domain MAE values $\left\{ {{\text{MAE}}_{\left( i \right)} } \right\}_{i = 1}^{N}$, as depicted in Fig. 2, using increasing coefficient IC values as defined by the following equation:

4 $$\begin{array}{*{20}c} {IC = \frac{1}{N - 1}\left| {\left\{ {{\text{MAE}}_{\left( i \right)} | {{\text{MAE}}_{{\left( {i + 1} \right)}} } > {\text{MAE}}_{\left( i \right)} } \right\}} \right| ,} \\ \end{array}$$

where a higher IC corresponds to a higher degree of monotonicity. Surprisingly, the IC values were relatively low, being 0.41, 0.63, 0.50, and 0.46 for the SNUMAT-MEGNet, C2DB-GPR-CFID, QMOF-KRR-ElemStat, and OMDB-RF-PLMF use cases, respectively. Despite the above trend in increasing in-domain MAE values with expanding applicability domain, nearly half of the entities in the $\left\{ {{\text{MAE}}_{\left( i \right)} } \right\}_{i = 1}^{N}$ sequences were less than their predecessors. IC as a local feature helped to capture the data noise, but it was not valuable for defining the global ordering of structures according to the applied UQ measure. Next, we calculated the ranking-based metric for the area under the confidence-oracle error (AUCO):

5 $$\begin{array}{*{20}c} {AUCO = \mathop \sum \limits_{i = 1}^{N - 1} \left( {{\text{MAE}}_{\left( i \right)}^{conf} - {\text{MAE}}_{\left( i \right)}^{orac} } \right),} \\ \end{array}$$

where ${\text{MAE}}_{\left( i \right)}^{conf}$ and ${\text{MAE}}_{\left( i \right)}^{orac}$ were the MAEs calculated based on the subsets of structures where the $i$ structures with the highest approximate (Δ-metric) and true (absolute error) UQ measure were removed, respectively. The corresponding $\left\{ {{\text{MAE}}_{\left( i \right)}^{conf} } \right\}_{i = 1}^{N - 1}$ and $\left\{ {{\text{MAE}}_{\left( i \right)}^{orac} } \right\}_{i = 1}^{N - 1}$ confidence curves were normalized to the $\left[ {0, 1} \right]$ range (Fig. 3), where a lower AUCO corresponded to a higher-ranking capability. It was possible to compare Δ-metric with other UQ strategies with the confidence curves in the unified form. Specifically, we considered the following methods. In the SNUMAT-MEGNet use case, a deep ensemble^81^ was implemented. The outputs of ten MEGNet models differing only in their initial weights were averaged, and the corresponding standard deviations served as the UQ measure. In the C2DB-GPR-CFID use case, the predictive variance naturally provided by GPR^82^ was taken into consideration. In the QMOF-KRR-ElemStat use case, we used the subsampling^83^ technique. Thirty KRR models were trained on 50% of the initial training set randomly sampled. The predictions on the test set were averaged, and the corresponding standard deviations served as the UQ measure. In the OMDB-RF-PLMF use case, the infinitesimal jackknife variance^84^ was employed for UQ. As shown in Fig. 3, the deep ensemble had a significantly smaller AUCO than the Δ-metric. In all other use cases, the suggested UQ criterion outperformed the competitive methods. It should be emphasized that UQ via the deep ensemble strategy required resource-intensive calculations associated with training multiple models, especially with advanced neural network architectures. As a result, the Δ-metric could act as a low-cost alternative with lower accuracy, but it could be readily applied to the UQ of a single model.

Strictly speaking, the above four UQ methods served to derive epistemic uncertainty, whereas the absolute observable error incorporated into the Δ-metric also included aleatoric uncertainty^85,86^. The former was the uncertainty related to the approximate predictive model, while the latter captured the noise inherent in the data. Scalia et al.^50^ demonstrated that epistemic uncertainty was the main contributor to the total uncertainty in the case of molecular data sets derived from the electronic structure theory. These data were typically self-consistent and characterized by low internal variability, i.e., low aleatoric uncertainty. Therefore, we concluded a provided comparison of the total uncertainty measure (Δ-metric) versus epistemic uncertainty techniques (deep ensemble, GPR variance, subsampling, and infinitesimal jackknife variance) was still valuable for the DFT and GW-derived bandgaps. However, this trick could probably not be used for the experimentally obtained^87,88^ bandgaps.

### Predictive intervals estimation

**Figure 4:**
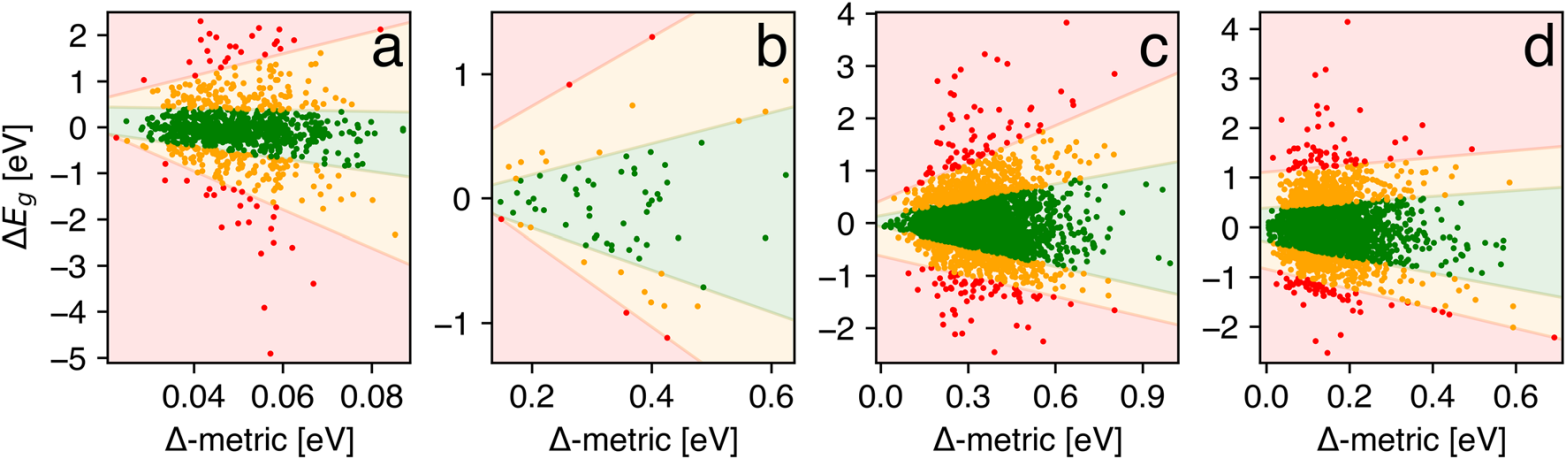
Predictive error as a function of the Δ-metric for the four considered use cases: (**a**) SNUMAT-MEGNet, (**b**) C2DB-GPR-CFID, (**c**) QMOF-KRR-ElemStat, and (**d**) OMDB-RF-PLMF. The colored areas correspond to the predictive intervals extracted by quantile regression: within one standard deviation (green), within two standard deviations (orange), and over two standard deviations (red).

After providing a general picture of the efficacy of the Δ-metric as a ranking criterion in UQ, we presented a strategy to compute the corresponding predictive intervals. An approximate form was required to transfer from the UQ measure to the predictive error. For instance, Janet et al.^38^ fitted the predictive variance to the conditional Gaussian distribution ${\mathcal{N}}\left( {0, \sigma_{1}^{2} + d\sigma_{2}^{2} } \right)$, where $d$ denotes the latent space distance (UQ measure), and $\sigma_{1}$ and $\sigma_{2}$ are the variable parameters. For the same purpose, we used quantile regression^89^. Given the errors in the test set and the corresponding Δ-metric values $\left\{ {\varepsilon_{i} ,{\Delta }_{i} } \right\}_{i = 1}^{N}$, the following optimization problem was solved by:

6 $$\begin{array}{*{20}c} {\mathop {\min }\limits_{{\beta \in {\mathbb{R}}^{p} }} \mathop \sum \limits_{i = 1}^{N} \rho_{\tau } \left( {\varepsilon_{i} - \xi \left( {{\Delta }_{i} ,\beta } \right)} \right) ,} \\ \end{array}$$

where $\xi$ is a parametric function of $\beta$, and $\rho_{\tau }$ is a tilted absolute value function for the $\tau$ quantile. For the sake of simplicity, $\xi$ was assumed a linear function of the parameters. We used quantiles that corresponded to one and two standard deviations, suggesting the normal distribution of errors (Fig. 4). As expected from previous analysis, the predictive intervals derived by quantile regression broadened significantly with increasing Δ-metric value, confirming its usefulness as a measure of predictive uncertainty. Guided by this illustrative representation of UQ measure-error dependence, one could define the cutoff Δ-metric value based on a desirable level of uncertainty in terms of the width of the predictive intervals.

The suggested strategy reproduced predictive intervals surprisingly well in terms of the quantiles. Thus, the model that was trained on 80% of the test data and examined the remaining 20% predicted the fraction of points within one standard deviation and within errors of 0.15, 2.38, and 1.47% in the SNUMAT-MEGNet, QMOF-KRR-ElemStat, and OMDB-RF-PLMF use cases, respectively. In the C2DB-GPR-CFID use case, where the test set contained just 72 points, the quantile regression model had a remarkably higher error of 18.39% for the same task. For instance, the 3 UQ methods considered by Tavazza et al.^37^ reached an error of about 10% in most cases.

### Out-of-domain applications

**Figure 5:**
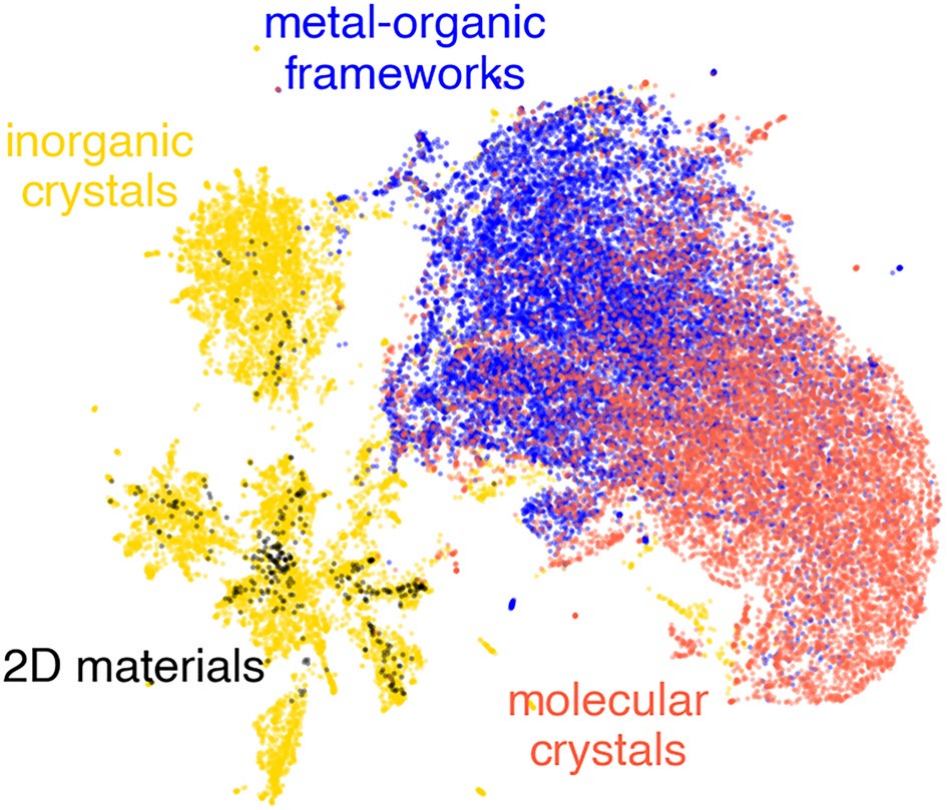
Two-dimensional representation of four considered materials subclasses extracted by the uniform manifold approximation and projection algorithm.

In the two previous sections, we used the subsets of the considered databases to verify the efficacy of the Δ-metric in UQ. Thus, a strong assumption that the training data and structures of interest sampled from the same distribution, i.e., independent and identically distributed, was made. In general, this was not the case^90^. Moreover, distinct classes of materials could form the former and latter distributions^91^. The performance of UQ measures in the out-of-domain regime has been of intense interest in advanced materials informatics applications, in particular, inverse design^92–95^ has been associated with the exploration of materials space beyond its well-known subregions. To model a scenario where the assumption of independent and identically distributed data was not satisfied, we applied implemented predictive models to predict the bandgap of materials from other data sets. Specifically, the MEGNet model trained on inorganic crystals was used to estimate the bandgap of two-dimensional materials (SNUMAT-MEGNet → C2DB-GPR-CFID), and the KRR model trained on metal–organic frameworks was tested on molecular crystals (QMOF-KRR-ElemStat → OMDB-RF-PLMF). Two-dimensional representations of SOAP-like descriptors obtained by the uniform manifold approximation and projection^96,97^ algorithm provided a first glimpse of the structural relationships between the donor and acceptor subsets (Fig. 5). The structures from the C2DB database appeared to be a nested subspace of the SNUMAT set of inorganic crystals. Indeed, approximately 9% of the structures from the SNUMAT database were identified as layered two-dimensional materials using the scoring parameter presented by Larsen et al.^98^ These layered compounds were hypothetical precursors for the monolayers that formed the C2DB database, where 21% of the considered C2DB structures had counterparts with identical chemical formulas among the two-dimensional SNUMAT compounds. Clouds of points corresponding to the metal–organic frameworks (QMOF-KRR-ElemStat) and molecular crystals (OMDB-RF-PLMF) partially overlapped.

**Figure 6:**
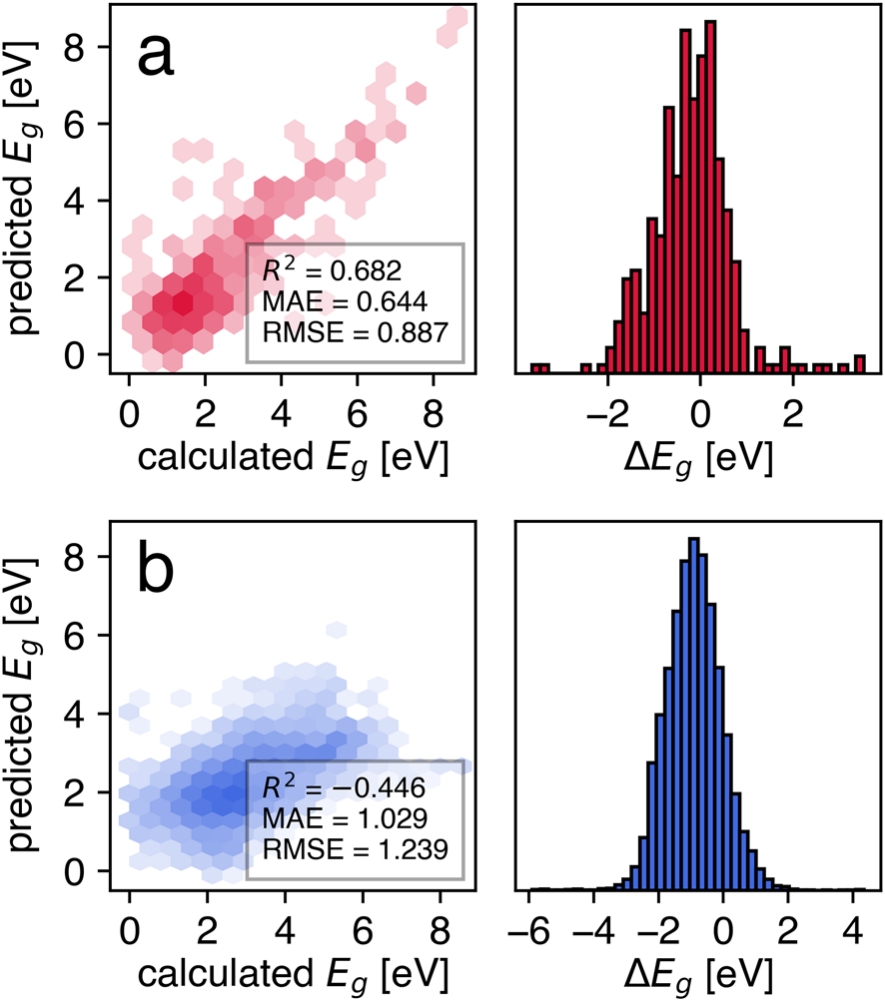
Parity plots and error histograms for the two considered out-of-domain use cases: (**a**) SNUMAT-MEGNet → C2DB-GPR-CFID, (**b**) QMOF-KRR-ElemStat → OMDB-RF-PLMF.

**Figure 7:**
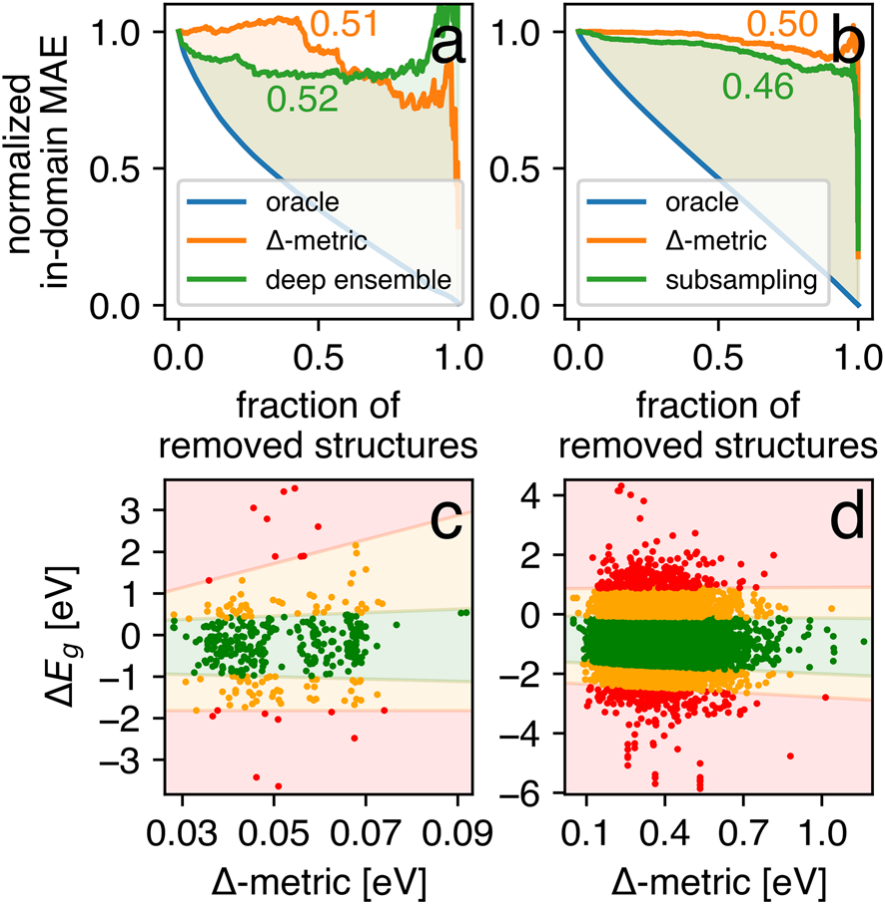
Confidence curves and predictive error as a function of the Δ-metric for the two considered out-of-domain use cases: (**a**, **c**) SNUMAT-MEGNet → C2DB-GPR-CFID, (**b**, **d**) QMOF-KRR-ElemStat → OMDB-RF-PLMF. Colored areas in subgraphs (**c**) and (**d**) correspond to the predictive intervals extracted by quantile regression: within one standard deviation (green), within two standard deviations (orange), and over two standard deviations (red).

A summary of MEGNet and KRR model performances in the out-of-domain applications is shown in Fig. 6. The MEGNet model demonstrated an acceptable level of accuracy in terms of MAE, RMSE, and the coefficient of determination (*R*^2^), whereas the KRR model trained on the MOFs did not give a practical estimation of the bandgap for molecular crystals. Surprisingly, this model was worse than a dummy predictor, whose output was the average ensemble value for every structure (*R*^2^ = 0.0, RMSE = 1.031, MAE = 0.798). The Δ-metric and the corresponding competitive UQ methods (deep ensemble and subsampling) showed comparable AUCO values of nearly 0.5 (Fig. 7), indicating a low ranking capability of all the above algorithms in the out-of-domain regime. Nevertheless, the Δ-metric helped to reduce in-domain MAE by 10 (20)% by considering 43 (25)% of the C2DB structures with the lowest UQ measure value. Similar behavior in the QMOF-KRR-ElemStat → OMDB-RF-PLMF use case was of little practical relevance because the reduced MAE value was still too high (greater than the error provided by a dummy predictor).

## Conclusions

In summary, we considered the performance of a new UQ measure in detail, which directly provided predictive intervals for individual model output in conjunction with quantile regression. Moreover, the Δ-metric made it possible to decrease the ensemble predictive error by choosing a proper subset of structures. In contrast to the well-known variational-inference-based methods, the proposed measure was directly applicable to the UQ of a single model and agnostic to the specific predictive algorithms and featurization schemes. We believed that the Δ-metric would also help explore new subregions of materials space beyond the assumption of independent and identically distributed data.

## Data Availability

All data used to train ML models are from the publicly available datasets: SNUMAT band gap dataset (https://www.snumat.com), Computational 2D Materials Database (https://cmrdb.fysik.dtu.dk/c2db), Quantum MOF database (https://materialsproject.org/mofs), and Organic Materials Database (https://omdb.mathub.io).

## Code availability

The code to calculate the presented UQ metric is available at https://github.com/korolewadim/dmetric.
